# Bis-3-Chloropiperidines Targeting TAR RNA as A Novel Strategy to Impair the HIV-1 Nucleocapsid Protein

**DOI:** 10.3390/molecules26071874

**Published:** 2021-03-26

**Authors:** Alice Sosic, Giulia Olivato, Caterina Carraro, Richard Göttlich, Dan Fabris, Barbara Gatto

**Affiliations:** 1 Department of Pharmaceutical and Pharmacological Sciences, University of Padova, Via Francesco Marzolo 5, 35131 Padova, Italy; alice.sosic@unipd.it (A.S.); giulia.olivato94@gmail.com (G.O.); caterina.carraro.2@phd.unipd.it (C.C.); 2 Institute of Organic Chemistry, Justus Liebig University Giessen, Heinrich-Buff-Ring 17, 35392 Giessen, Germany; Richard.Goettlich@org.Chemie.uni-giessen.de; 3 Departments of Chemistry and Biological Sciences, University at Albany-SUNY, 1400 Washington Avenue, Albany, NY 12222, USA; dan.fabris@uconn.edu

**Keywords:** TAR-RNA, bis-3-chloropiperidines, alkylating agents

## Abstract

Specific RNA sequences regulate functions essential to life. The Trans-Activation Response element (TAR) is an RNA stem–bulge–loop structure involved in several steps of HIV-1 replication. In this work, we show how RNA targeting can inhibit HIV-1 nucleocapsid (NC), a highly conserved protein known to catalyze nucleic acid melting and strand transfers during reverse transcription. Our RNA targeting strategy consists of the employment of bis-3-chloropiperidines (B-CePs) to impair RNA melting through bifunctional alkylation. Specific interactions between B-CePs and TAR RNA were analytically investigated by gel electrophoresis and mass spectrometry, allowing the elucidation of B-CePs’ recognition of TAR, and highlighting an RNA-directed mechanism of protein inhibition. We propose that B-CePs can freeze TAR tridimensional conformation, impairing NC-induced dynamics and finally inhibiting its functions in vitro.

## 1. Introduction

The field of RNA science has exploded in the past decade and is radically changing the understanding of the role that RNA plays in fundamental biological processes. Although RNA has been considered an “undruggable” pharmaceutical target for a long time, it is now accepted as a next-generation space for drug development.

**Figure 1:**
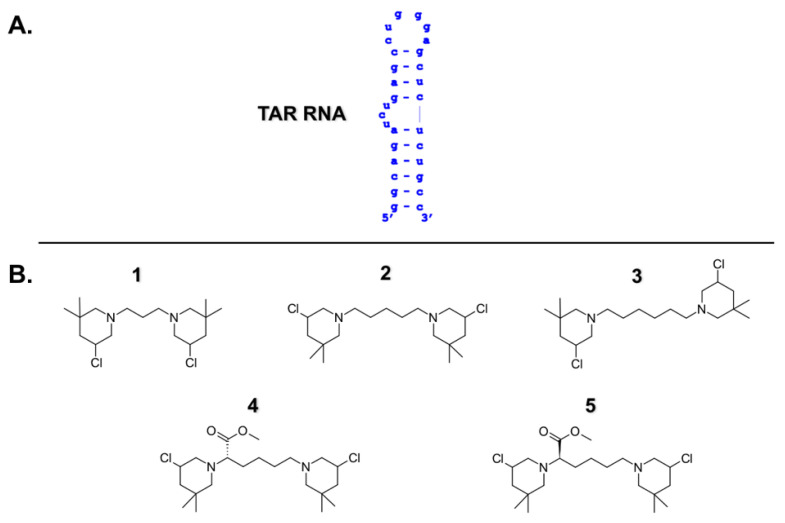
(**A**) Sequence and secondary structure of oligonucleotide-construct replicating trans-activation response (TAR) RNA which was employed in our assays. (**B**) Chemical structure of bis-3-chloropiperidines analyzed in this work.

A highly investigated and well characterized RNA structure is the HIV-1 trans-activation response element (TAR), an interesting target for the development of antiviral drugs [1,2,3,4,5,6,7]. TAR consists of a stable bulge–loop structure (Figure 1A) located at the 5′ end of the viral genome. The biologically relevant domain of TAR is the substrate of multiple viral RNA-binding proteins, playing a critical role in several biological processes within the viral replication cycle [8,9,10,11,12,13]. In particular, we became interested in TAR as the substrate of the HIV-1 nucleocapsid (NC) protein [14,15,16,17,18,19].

NC is a nucleic acid chaperone that, during reverse transcription, destabilizes the stable secondary structure of TAR-RNA and promotes its refolding into a more thermodynamically stable conformation to allow reverse transcription to proceed [20]. Based on its conservation among several HIV-1 strains, the inhibition of NC represents a promising strategy to develop new anti-HIV pharmacological treatments for overcoming drug resistance [20]. To this extent, we focused on the impairment of NC activities through small molecules acting as binders/stabilizers in its nucleic acid substrates, and thus identified 2,6-dipeptidyl-anthraquinones as potent in vitro NC inhibitors [14,17,19]. Structure-activity relationship studies of this class of threading intercalators demonstrated how their molecular mechanisms of enzymatic inhibition mainly rely on the stabilization of the nucleic acid substrates of NC [14,18,19]. Prompted by these findings, here we explore TAR-RNA targeting of another class of nucleic acid binders acting with a different mechanism from the intercalators.

Bis-3-chloropiperidines (B-CePs) are recently developed alkylating agents, thoroughly investigated for their mechanism of action toward DNA [21,22,23,24,25,26]. Interestingly, the analysis of a large library of B-CePs revealed that high in vitro reactivity toward DNA mirrors a poor cytotoxicity despite a good cell permeability [26]. The most potent B-CePs react fast with available nucleophiles before reaching the cell nucleus, resulting in lower genomic DNA damage, and higher susceptibility of competing reactions in the cytoplasmic environment. Given this observation, we reasoned that fast-reacting B-CePs may hide an unveiled RNA-targeting potential and could possibly disclose new candidates for alternative therapeutic applications. To test this hypothesis, we selected compound **1** as the B-CePs prototype, owing to its high reactivity and low cytotoxicity [22,25], and explored its ability to interact with TAR RNA. We enriched the set with compounds **2** and **3**, characterized by a longer alkylic linker connecting the reactive moieties, and with the lysine ester compounds **4** and **5** (Figure 1B), all of them showing a high reactivity toward nucleophiles [23,24,26]. The molecular details of B-CePs interaction with TAR led us to propose them as putative agents for RNA targets with therapeutic potential for inhibiting HIV-1 NC-mediated effects.

## 2. Results and Discussion

### 2.1. B-CePs Stabilize TAR RNA Hairpin Structure

**Figure 2:**
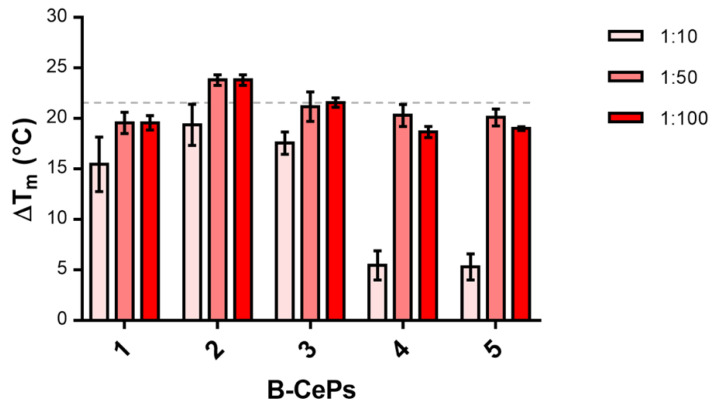
Variations of TAR RNA melting temperature (ΔT_m_) in the presence of an increasing concentration of B-CePs **1**–**5**. Reported values are the mean ± standard error of the mean (SEM) of triplicate experiments performed on samples containing 1 μM of oligoribonucleotide and 10, 50 and 100 μM of compound. TAR RNA was folded in a BPE buffer and an incubation of TAR RNA with each compound at 37 °C for 2 h was performed before the analyses. Reference the T_m_ value for TAR RNA in the absence of compound is 52.9 °C.

A first insight into the putative interaction of B-CePs with RNA was offered by the investigation of the ability of the selected compounds to stabilize the TAR hairpin structure. We employed fluorescence quenching assays (FQA) to measure the ability of each B-CeP to modify the thermal denaturation profile of the TAR construct shown in Figure 1A, which was functionalized at both termini with a fluorophore-quencher couple [14,19]. In this way, thermal melting of the structure can be assessed from the intensity of the detected fluorescence signal. The melting profile of TAR in the presence of increasing concentrations of each compound allowed an evaluation of the shift in melting temperature (ΔT_m_) from the reference value, which was determined in the absence of compound. Sample solutions contained 1 μM of RNA substrate and 10, 50 or 100 μM of each B-CePs compound. Figure 2 summarizes the values of ΔT_m_ obtained by adding increasing concentrations of B-CePs **1**–**5**.

Assuming that the T_m_ variation is directly correlated with the RNA-binding activity of the compound, the results clearly revealed that the tested compounds are efficient stabilizers of the secondary structure of TAR RNA. Figure 2 allows an initial evaluation of the tested set. At 10 μM, the T_m_ increase was particularly significant in the presence of B-CePs **1**–**3**, whereas derivatives **4** and **5** produced a much weaker stabilization effect. Interestingly, at 50 μM, all compounds achieved a similar efficient stabilization with an averaged ΔT_m_ of 21 ± 2 °C and did not raise when increasing the compound concentration to 100 μM, suggesting that the interaction of B-CePs with TAR reached completion in a narrow range of doses. These results show evidence of the recognition between B-CePs and a hairpin-structured RNA construct and prompted us to proceed in disclosing additional binding details.

### 2.2. B-CePs Directly Interact with TAR RNA

**Figure 3:**
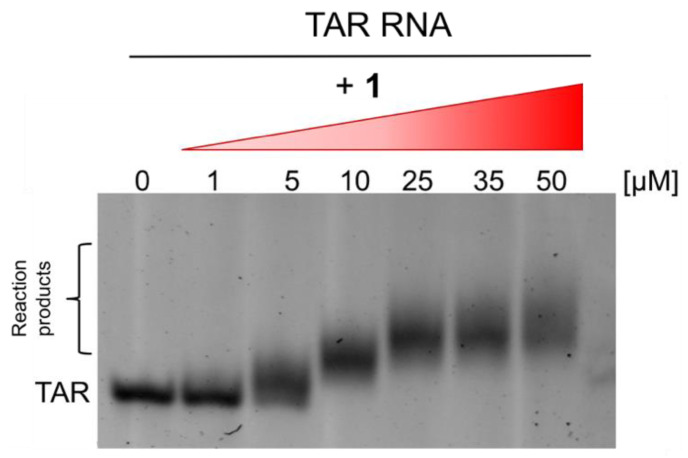
Non-denaturing polyacrylamide gel electrophoresis (PAGE) (PAA 12%) in TBE 1× showing the concentration-dependence of the interaction of B-CeP **1** with TAR. Folded TAR hairpin 1 μM was incubated with increasing concentrations of compound (0, 1, 5, 10, 25, 35, 50 μM) for 2 h at 37 °C with a BPE buffer (pH 7.4).

To further evaluate the interaction of B-CePs to TAR RNA, Electrophoretic Mobility Shift Assay (EMSA) was used as a simple tool to visualize the putative interaction of compound **1** with TAR. The RNA was folded in its hairpin secondary structure, incubated for 2 h at 37 °C with increasing concentrations of compound **1** (0, 1, 5, 10, 25, 35, 50 μM), and the reaction products were resolved by polyacrylamide gel electrophoresis (PAGE). Figure 3 provides the results obtained in non-denaturing PAGE.

The electrophoretic mobility of the RNA substrate was clearly altered by the presence of the B-CeP. As shown in Figure 3, we observe a band slightly smeared up at 1 μM compound, corresponding to the 1:1 compound/substrate molar ratio. This migration delay is absent in the untreated control. Increasing the compound concentration, the band clearly shifts up in a dose-dependent manner, suggesting the formation of multiple stable products. Binding events reach a plateau at 25 μM, with the band shift maintained at a constant up to the highest tested concentration (50 μM). Interestingly, the shifted bands are very stable in the gel conditions, differently from what we observed when analyzing non-covalent binding between intercalators and TAR in similar gel systems [14,17,18].

### 2.3. B-CePs Covalently React with TAR RNA

**Figure 4:**
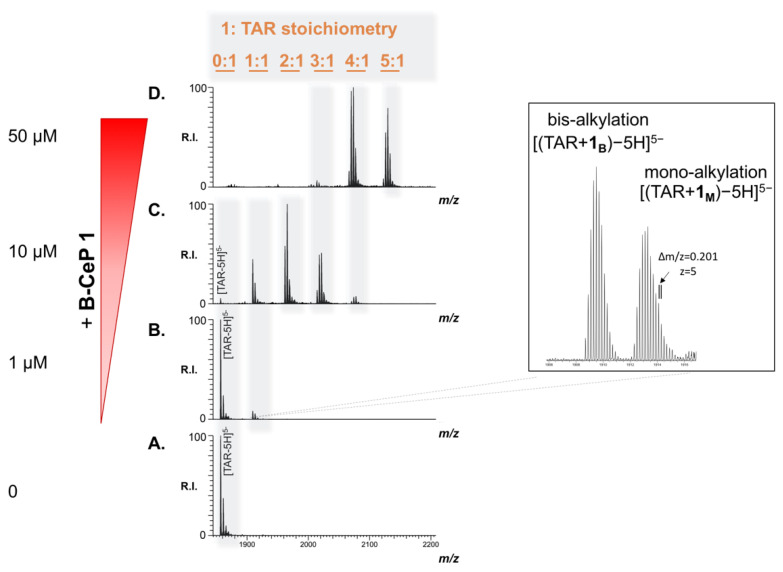
Representative electrospray-ionization mass spectrometry (ESI-MS) spectra of reaction mixtures obtained by incubating TAR (1 μM) with increasing B-CeP **1** (**A**:0, **B**:1, **C**:10, **D**:50 μM) at 37 °C for 2 h in a BPE buffer (pH 7.4). The spectra **B**–**D** show the concentration-dependent reactions induced by B-CeP **1** on TAR. Greater stoichiometries corresponding to combinations of mono- and bi-functional adducts, were detected and indicated in orange in the figure. The spectra were recorded in 150 mM ammonium acetate. Lower intensity signals near free/adducted species consist of typical sodium and ammonium adducts. Only region containing the 5-charge state is shown for the sake of clarity.

To elucidate the molecular details of the products observed by PAGE, we further characterized the outcome of the reaction with TAR by electrospray ionization-mass spectrometry (ESI-MS). A preliminary determination was accomplished on the individual TAR construct, and a representative ESI-MS spectrum is provided in Figure 4A. Only the region containing the 5-charge state is shown for the sake of clarity. The detected species labelled [TAR-5H]^5−^ in Figure 4A afforded an experimental mass of 9286.25 Da, which matches the monoisotopic value of 9286.24 Da calculated from the RNA sequence very closely (Table 1). Successively, we performed the experiment by reacting compound **1** with TAR at 1 μM. After 2 h of incubation, at 37 °C, the reaction mixture was submitted to ethanol precipitation to achieve quenching and desalting, which enabled a direct infusion ESI-MS analysis. The representative ESI-MS spectrum of the 1:1 compound/substrate molar ratio provided in Figure 4B shows new peaks, in addition to an intense signal corresponding with the unreacted TAR. The new species, which displayed a greater mass compared to the individual TAR, did not correspond to the sum of substrate-ligand masses expected by non-covalent interactions. Rather, the new products detected were readily assigned to covalent adducts upon reaction of compound **1** with TAR, which could be due to alkylation at specific sites. This hypothesis is based on analogies to the mechanism of bis-3-chloropiperidines reaction with DNA, which have been demonstrated to proceed through a reactive bicyclic aziridinium intermediate formed upon intramolecular nucleophilic displacement of chloride by nitrogen. In the presence of nucleic acids, aziridinium preferentially attacks N7 of guanines on the substrate, forming different types of adduct (Scheme S1 in Supplementary Materials).^22^ Analyzing a new species in Figure 4B, their masses matched those of products generated by either mono- or bis-alkylation reactions. The species TAR + **1_B,_** displaying a mass of 9548.49 u, corresponded to bi-functional adducts formed by the reaction of both 3-chloropiperidine reactive moieties with the RNA strand (labelled [TAR + **1_B_** − 5H]^5−^ in the inset in Figure 4B). Differently, TAR + **1_M_** species with a mass of 9566.49 u was assigned to mono-functional adducts, in which only one of the 3-chloropiperidines reacted with the substrate, whereas the other was hydrolyzed to 3-hydroxyl by the aqueous environment (labelled [TAR + **1_M_** − 5H]^5−^ in the inset in Figure 4B). Table 1 summarizes the experimental and calculated masses of the new products and provides the assignment of the various species detected in Figure 4B.

The results shown in Figure 4C,D show the reaction products obtained, further increasing the concentration of compound **1**, with 1 μM TAR solution incubated with 10 and 50 μM of B-CeP **1,** respectively. Direct ESI-MS analysis of the reaction mixtures allowed for the identification of multiple additional products: different combinations of mono- and bi-functional covalent adducts were readily recognized based on their mass shift from the unmodified substrate. Incremental binding stoichiometries up to five equivalents of compound **1** were detected as compound concentration increased, consistently with the trend observed in the gel system in Figure 3. Differently from the reactivity exhibited by compound **1** toward DNA, which produced extensive backbone cleavage [22,25] in the case of TAR RNA the fragmentation of the oligoribonucleotide chain was not detected by ESI spectrometry, or within the gel electrophoresis systems.

Similar results were obtained when we employed ESI-MS to evaluate the ability of B-CePs **2**–**5** to react with the TAR construct (Figure S1). All the compounds were able to produce stable alkylation adducts with multiple stoichiometries consisting of different combinations of mono- and bi-functional products. Moreover, these assays also allowed us to calculate the percentage of adducted substrates over the total RNA in each sample and the results are reported in Figure S1. Hence, we conclude that B-CePs react with the stem–loop structure of TAR RNA producing stable mono- and bi-functional alkylation products.

### 2.4. Adducts Mapping on TAR RNA

**Figure 5:**
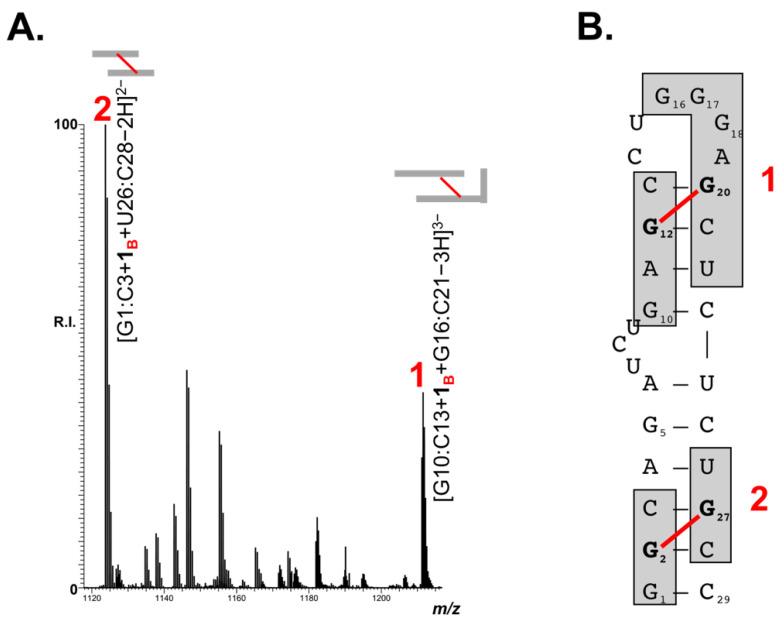
(**A**) Representative region of ESI-MS spectrum obtained after RNAse A digestion of TAR RNA (1 μM) reacted with 10 μM of B-CeP **1**. Only the most relevant bridged alkylation products, which are cross-referenced in Table 2 and highlighted in the cartoon on panel B, are indicated in the spectrum. Oligonucleotides products are indicated by the first and last base separated by colon. Product labels indicate the bi-functional alkylation products bridging base-paired regions within the TAR RNA hairpin. (**B**). Cartoon of the B-CeP **1**-induced bi-functional alkylation products bridging base-paired regions within the TAR RNA secondary structure.

The ability of B-CePs to covalently and efficiently react with TAR and the observed marked stabilization of its hairpin structure hints that the adducts may be favorably located to prevent stem melting. To identify the position of the B-CePs adducts within the folded TAR construct, we employed a bottom-up approach: the strategy is based on RNAse A digestion after the TAR reaction with B-CeP **1**, followed by ESI-MS identification of the relevant hydrolytic products. The TAR control, treated with the endonuclease, revealed, as expected, the formation of multiple RNA fragments, listed in Table S1. According to RNAse A specificity, which hydrolyzes preferentially single-stranded C and U, the detected fragments corresponded to stretches of TAR cleaved only after pyrimidine residues. The TAR-**1** reaction mixture, corresponding to the conditions shown in Figure 4C, was then assayed. The results obtained upon RNAse A digestion allowed the identification of multiple alkylated products, as shown in the representative region of ESI-MS spectrum reported in Figure 5A. The new species, bearing either mono- or bi-functional adducts, matched RNA fragments positioned in both stem and loop regions of the TAR structure. The majority of fragments obtained upon the B-CeP **1** treatment, reported in Table S2, were also identified in their non-alkylated form in the control experiment (Table S1), with the notable exception of two new distinctive fragments. The first fragment, labelled **1** in Figure 5, has a mass of 3636.73 u and matches the sequences of G10:C13 and G16:C21 bridged by a bi-functional adduct. In the folded TAR hairpin, the two stretches are partially complementary as a result of the base-pairing of three nucleotides. In the untreated TAR control (Table S1), the two fragments were identified as individual species, as they easily denature under the employed ESI-MS experimental conditions. In the B-CeP-treated control, on the other hand, the bifunctional adduct is strategically located to bridge the G10:C13 fragment to the G16:C21, thus stabilizing the two digestion products into the single detected conjugate G10:C13 + **1_B_** + G16:C21. Accordingly, the second new species identified, labelled **2** in Figure 5, with a mass of 2249.52, was ascribed to the fragments G1:C3 and U26:C28 bridged by compound **1** into G1:C3 + **1_B_** + U26:C28. The two bridged alkylation products are visualized on the secondary structure of TAR RNA in Figure 5B and their features are summarized in Table 2.

It should be noted that the short base-paired region in **1** is characterized by the presence of a base-paired GC step (G12–C13 complementary to G20–C21), which places G12 in close spatial proximity with G20. Considering that Gs are the most susceptible nucleophilic center in nucleic acids (1), we assigned G12 and G20 as the putative nucleotides engaged in the bifunctional product connecting the two strands (Figure 5B). Once again in the species **2** (G1:C3 + **1_B_** + U26:C28), the presence of the two guanine residues, G2 and G27, opportunely placed to be bridged by compound **1**, allowed us to assume the putative location of the bifunctional adduct in the species **2,** as shown in Figure 5B.

Hence, the accurate MS-analysis of the species obtained after nuclease footprinting of TAR RNA construct reacted with B-CeP **1** unambiguously revealed the presence of alkylation products able to covalently link opposing strands within TAR RNA hairpin structure. These adducts, formed upon reaction with B-CePs, are likely those mainly responsible for the stabilization of the TAR secondary structure, preventing its melting, as observed in the experiments shown in Figure 2.

### 2.5. B-CePs Inhibit NC-Mediated Melting of TAR RNA In Vitro

Having demonstrated that B-CePs bind and stabilize the RNA substrate of NC, we performed a high-throughput screening (HTS) to analyze the in vitro ability of the tested B-CePs to inhibit NC-mediated melting of the TAR RNA hairpin construct [14,16,17,18,19]. This assay is based on the doubly labeled TAR construct used above in the FQA experiments and is extensively employed to screen for inhibitors of NC melting activity [27,28]. After preliminary controls to ensure the absence of direct quenching activity by the compounds under investigation, their ability to inhibit TAR RNA melting was evaluated in the presence of recombinant full-length HIV-1 NC. By adding increasing amounts of B-CePs, we determined the concentration of each compound that induced a 50% reduction in the destabilizing activity of NC (i.e., IC_50_) on TAR. The results are reported in Table 3.

Clearly, all tested B-CePs were shown to be potent inhibitors of NC-induced melting of TAR in vitro, exhibiting IC_50_ values < 5 μM. In good agreement with the thermal melting results reported above, compound **2** is also slightly more potent in NC inhibition. Interestingly, compound **2** was also the most reactive towards the TAR construct, as seen by the comparison of the ESI-MS spectra obtained at the same B-CeP/TAR molar ratio (Figure S1). Melting stability and alkylation efficiency are related, although the higher potency in alkylation of **1** vs. **3** (Table S1) leads also to a wider range of RNA adducts, including those probably not affecting the helix melting. The optimal compromise between melting inhibition and alkylation potency is seen in compound **2**. For this compound, our results nicely support the correlation between B-CePs reactivity toward TAR, TAR stabilization and potent in vitro inhibition of the NC-mediated melting. Unfortunately, the increase in the length of the linker in compound **2** also increases cell permeability and cytotoxicity [26], making it less desirable as a putative antiviral compound.

Finally, we tested the possibility that B-CePs may be capable of binding directly to the NC protein. Experiments were carried out by mixing NC with B-CePs **1**–**5**, and then analyzing the samples using ESI-MS under non-denaturing conditions. The results showed unambiguously that the test compounds were not capable of binding directly to the NC protein (data not shown), thus indicating that their ability to interfere with NC was subordinate to their remarkable TAR-binding properties.

## 3. Materials and Methods

### 3.1. RNA Substrate and Protein

TAR is the 29-mer RNA sequence 5′ GGCAGAUCUGAGCCUGGGAGCUCU CUGCC 3′ and was synthesized by Metabion International AG (Martinsried, Germany). Stock solutions were stored at −20 °C in 10 mM Tris-HCl, pH 7.5. Dilutions were made in DEPC-treated water (Ambion). When specified, TAR was labeled at 5′ and 3′ ends, respectively, by the fluorophore 5-carboxyfluorescein (FAM) and the dark quencher 4-(4′-dimethylaminophenylazo)benzoic acid (Dabcyl). The typical folding procedure consisted of snap-cooling: TAR RNA diluted in the proper aqueous buffer was heated to 95 °C for 5 min and then ice-cooled in order to assume the proper hairpin structure.

The full-length recombinant NC protein was obtained as reported [29]. The protein concentration was determined on a UV−vis Spectrophotometer Lambda 20, PerkinElmer (Waltham, MA, USA) using an extinction coefficient at 280 nm of 6410 M^−1^ cm^−1^.

### 3.2. Chemical Reagents

Bis-3-chloropiperidines **1**–**5** (Figure 1B) were synthesized in house, as previously described [23,24]. Aliquots of chemical probes were freshly prepared by diluting an 8 mM DMSO stock in MilliQ water and were instantly reacted with the RNA substrate to avoid the typical quenching effects of the aqueous environment. All the other chemical reagents, including salts and solvents, were purchased from Sigma-Aldrich (Milan, Italy).

### 3.3. Fluorescence Quenching Assay (FQA)

The ability of the tested compounds to stabilize the TAR RNA structure was measured by the increase in the melting temperature of the oligoribonucleotide in the presence of different concentrations of each compound. Melting temperature (T_m_) is the temperature at which 50% molecules of oligoribonucleotide are denatured. TAR RNA hairpin was formed by snap cooling in 1× BPE buffer (NaH_2_PO_4_ 0.2 mM, Na_2_HPO_4_ 0.6 mM, Na_2_EDTA 0.1 mM) as described above. Prior to analysis, TAR RNA was incubated with different concentrations (final concentrations of 10, 50, and 100 μM) of each compound at 37 °C for 2 h. Nucleic acid solutions without compound were used to measure the reference T_m_ value. The melting protocol consisted of a melting phase, in which the temperature increased from 25 to 99 °C in 1 h (0.02 °C/s). Fluorescence emission of FAM was read by using a Light Cycler 480 II (Roche, Basel, CH) with emission at λ = 510 nm and correlated to the melting temperature of the oligoribonucleotide. The T_m_ value was mathematically derived from the thermal denaturing profile by using LC480 software. ΔT_m_ was calculated by using the following equation: ΔT_m_ = T_m2_ − T_m1_, where T_m2_ and T_m1_ are the T_m_ values measured by testing the RNA structure in the presence or absence of compound, respectively.

### 3.4. Gel Electrophoretic Analysis

Electrophoretic Mobility Shift Assay (EMSA) was used to assess the ability of compound **1** to directly interact to TAR RNA construct. Prior to incubation with compound, TAR RNA was heated to 95 °C for 5 min and then ice-cooled in order to assume the proper hairpin structure. TAR RNA construct (1 μM) was then incubated with increasing concentrations (from 0 to 50 μM) of B-CeP **1**, at 37 °C for 2 h in 1× BPE buffer (pH 7.4). After incubation, products were resolved by 12% non-denaturing polyacrylamide (PAA) gels containing 1× TBE (Tris Borate EDTA) buffer at room temperature. Unreacted and reacted RNA on the gel were stained with SybrGreen II^®^ (Invitrogen, Carlsbad, CA, USA). Fluorescence in gel system was detected on a Geliance 600 Imaging System (PerkinElmer, Waltham, MA, USA).

### 3.5. Mass Spectrometric Analysis

Samples containing a final 1 μM concentration of TAR RNA in 1× BPE buffer were added with compound 1 at final concentrations of 1, 10, 50 μM, depending on the pur- pose of the experiment. Reaction mixtures were incubated at 37 °C for 2 h. Samples prepared in BPE were buffer-exchanged by performing ethanol precipitation in the presence of 1 M of ammonium acetate. In case of reaction mixtures, the treatment also served to achieve reaction quenching. Samples were re-dissolved and diluted in 150 mM ammonium acetate (pH adjusted to 7.0) to achieve a final 1 μM concentration of total RNA. All samples were analyzed by direct infusion electrospray ionization (ESI) on the Thermo Fisher Scientific (West Palm Beach, CA, USA) LTQ-Orbitrap Velos mass spectrometer. The analyses were performed in nanoflow mode by using quartz emitters produced in-house by using a Sutter Instruments Co. (Novato, CA, USA) P2000 laser pipette puller. Up to 5 μL samples were typically loaded onto each emitter by using a gel-loader pipette tip. A stainless steel wire was inserted in the back-end of the emitter to supply an ionizing voltage that ranged between 0.8 and 1.2 kV. The source temperature and desolvation conditions were adjusted by closely monitoring the incidence of ammonium adducts and water clusters. Control determinations were completed to verify the experimental masses of the TAR RNA substrate. Data were processed by using Xcalibur 2.1 software (Thermo Scientific, West Palm Beach, CA, USA).

### 3.6. Enzymatic Digestion of TAR RNA

The TAR RNA was heated to 95 °C for 5 min and then ice-cooled in order to assume the proper hairpin structure. Typical reactions consist of a final 1 μM solution of TAR RNA in 1× BPE buffer with compound **1** at final concentrations of 10 μM, i.e., a 10:1 compound/substrate ratio. Reaction mixtures were incubated at 37 °C for 2 h and quenched by ethanol precipitation. Aliquots of unreacted and reacted TAR were submitted to digestion with ribonuclease A (RNAse A) in 150 mM ammonium acetate for 1 h at 37 °C. Samples were stored at −20 °C until immediate analysis by ESI-MS, as reported in the paragraph above.

### 3.7. High-Throughput Screening (HTS)

High-throughput screening (HTS) was performed to identify the inhibitors of NC chaperone activity on TAR. We used a VictorIII (PerkinElmer) microplate reader with 485 and 535 nm as the excitation and emission wavelengths. The TAR RNA hairpin was formed by snap cooling in the 1× BPE buffer, as described above. A 1 μM aliquot of TAR, which bore 5′-FAM and 3′-DAB modifications, was folded in 1× BPE buffer. The samples were then diluted to 0.1 μM in 1× BPE buffer. Increasing concentrations of compound (0, 0.1, 0.5, 1, 5, 10, 50, 100 μM final) were added to each sample and incubated at 37 °C for 2 h before introducing NC to a final concentration of 0.8 μM (for a 1:8 oligo to NC molar ratio). The plate was immediately read at room temperature three times with 1 min intervals, unless differently specified. The experimental data were fitted as reported earlier to enable calculation of the respective IC_50_ value.16 Each experiment was performed in triplicate to calculate an average and standard deviation.

## 4. Conclusions

Bis-3-chloropiperidines represent a recently developed class of nucleic acid reactive agents. Beyond their expected ability to induce DNA cleavage, we reveal in this work B-CePs reactivity with a hairpin structured RNA, such as the HIV-1 TAR domain, which is a substrate of several enzymatic activities relevant for HIV-1 replication. Considering the different conformations of double-stranded regions of RNA and DNA, we anticipate different effects ascribed to the tertiary structure of the helix. To shed more light on this important structural issue, a thorough analysis of B-CePs reactivity toward models of single- and double-stranded RNA substrates is underway.

B-CePs covalently react with the TAR hairpin, producing peculiar bifunctional adducts bridging the stem portion of TAR construct and leading to its strong stabilization. Analogously to the mechanisms of NC inhibition previously observed for 2,6-dipeptidyl anthraquinones acting as threading intercalators, stabilization of TAR by B-CePs leads to the impairment of NC destabilizing activity in vitro. However, differently from the anthraquinone derivatives, which were also able to directly interact with NC protein, the mechanism of NC inhibition by B-CePs totally relies on their TAR targeting, thus confirming an extraordinary tropism for nucleic acids.

Hence, the employment of agents able to react with the RNA partners of NC blocking structure remodeling represents an original strategy that draws the spotlight on the RNA-mediated processes. The proposed strategy may represent a springboard for the development of a new class of RNA-targeting therapeutics to fight old and new disease associated with RNA viruses.

## Figures and Tables

**Table 1:** Name, description, experimental and calculated masses for the species detected in Figure 4B [a].

Name	Description	Mass (u) [b]	
		Experimental	Calculated
TAR	TAR oligoribonucleotide construct	9286.25	9286.24
TAR + **1_B_**	bis-alkylation product of 1 on TAR	9548.49	9548.48
TAR + **1_M_**	mono-alkylation product of 1 on TAR	9566.49	9566.48

[a] These products were obtained by reacting TAR (1 μM) with B-CeP **1** (1 μM) at 37 °C for 2 h. [b] Monoisotopic masses are reported in mass units (u).

**Table 2:** Bi-functional alkylation products bridging base-paired regions within TAR RNA hairpin detected by treating TAR RNA with B-CeP **1**, followed by RNAse A digestion. Product labels refer to Figure 5. Oligonucleotides products are indicated by the first and last base, separated by colon. Product labels are also reported on the hairpin secondary structure cartoon in Figure 5B.

Label	Bridged RNA Fragments	Symbol	Exp. Mass (u)	Calc. Mass (u)
**1**	G10:C13 + **1_B_** + G16:C21	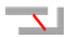	3636.73	3636.73
**2**	G1:C3 + **1_B_** + U26:C28	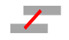	2249.52	2249.52

**Table 3:** Inhibition of NC-mediated melting of individual TAR RNA hairpin by B-CePs.

B-CePs	Linker	IC_50_^a^ [μM]
**1**	-(CH_2_)_3_-	4.75 ± 0.15
**2**	-(CH_2_)_5_-	2.75 ± 0.70
**3**	-(CH_2_)_6_-	3.55 ± 0.37
**4**	-(CH_2_)_5_COOCH_3_	5.09 ± 0.05
**5**	-(CH_2_)_5_COOCH_3_	4.78 ± 0.71

^a^ Values are the mean of data obtained from three experiments, each performed in triplicate.

## Supplementary Materials

The following are available online, Scheme S1: Structure of the bicyclic aziridinium intermediate formed upon intramolecular nucleophilic displacement of chloride by nitrogen. Figure S1: Representative ESI-MS spectra of reaction mixtures obtained by incubating TAR (1 μM) with 10 μM of different B-CePs at 37 °C for 2 h in BPE buffer (pH 7.4). Table S1: Digestion products obtained by treating TAR RNA control with RNAse A. Table S2: Digestion products obtained by treating B-CeP **1**-reacted TAR RNA with RNAse A corresponding to unbridged RNA fragments.

## Sample Availability

Samples of the compound **1** are available from the authors.
