# Supplementary material for: Bis-3-Chloropiperidines Targeting TAR RNA as A Novel Strategy to Impair the HIV-1 Nucleocapsid Protein
Source: Molecules. 2021 Mar 26;26(7):1874. doi: 10.3390/molecules26071874 (PMC8038054; doi:10.3390/molecules26071874)
Supplement: Supplementary file 1 [file molecules-26-01874-s001.pdf]

Supporting Information

Bis-3-chloropiperidines targeting TAR RNA as a novel strategy to impair the HIV-1 nucleocapsid protein

Alice Sosic [1], Giulia Olivato [1], Caterina Carraro [1], Richard Göttlich [2], Dan Fabris [3], Barbara Gatto [1*]

[1] Department of Pharmaceutical and Pharmacological Sciences, University of Padova, Via Francesco Marzolo 5, 35131 Padova (Italy); alice.sosic@unipd.it (A.S.), giulia.olivato94@gmail.com (G.O.), caterina.carraro.2@phd.unipd.it (C.C.), barbara.gatto@unipd.it (B.G.)

[2] Institute of Organic Chemistry, Justus Liebig University Giessen, Heinrich-Buff-Ring 17, 35392 Giessen (Germany); Richard.Goettlich@org.Chemie.uni-giessen.de (R.G.)

[3] Departments of Chemistry and Biological Sciences, University at Albany-SUNY, 1400 Washington Avenue, Albany, NY 12222 (USA); dan.fabris@uconn.edu (D.F.). Present Address: Dan Fabris, Department of Chemistry, University of Connecticut, Storrs, CT, 06269, USA

* Correspondence: barbara.gatto@unipd.it (B.G.)

Table of content

Scheme S1 **p. 2**

Figure S1 **p. 3**

Table S1 **p. 4**

Table S2 **p. 4**

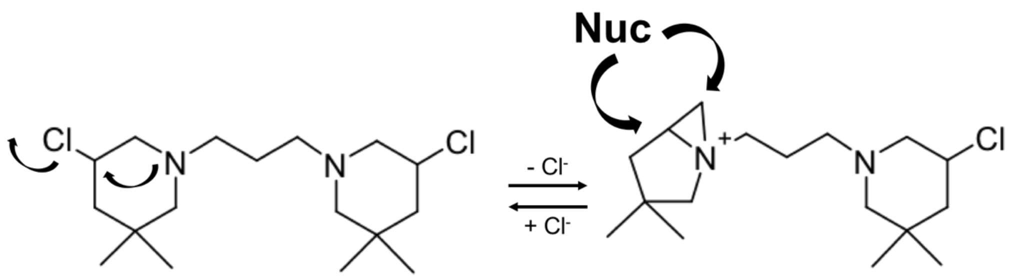

Scheme S1. Structure of the bicyclic aziridinium intermediate formed upon intramolecular nucleophilic displacement of chloride by nitrogen. The reactive aziridinium ion can be readily attacked by nucleophiles (Nuc). The electrophilic bicyclic aziridinium intermediate can be attacked by nucleophilic sites present on either DNA/RNA or solvent.

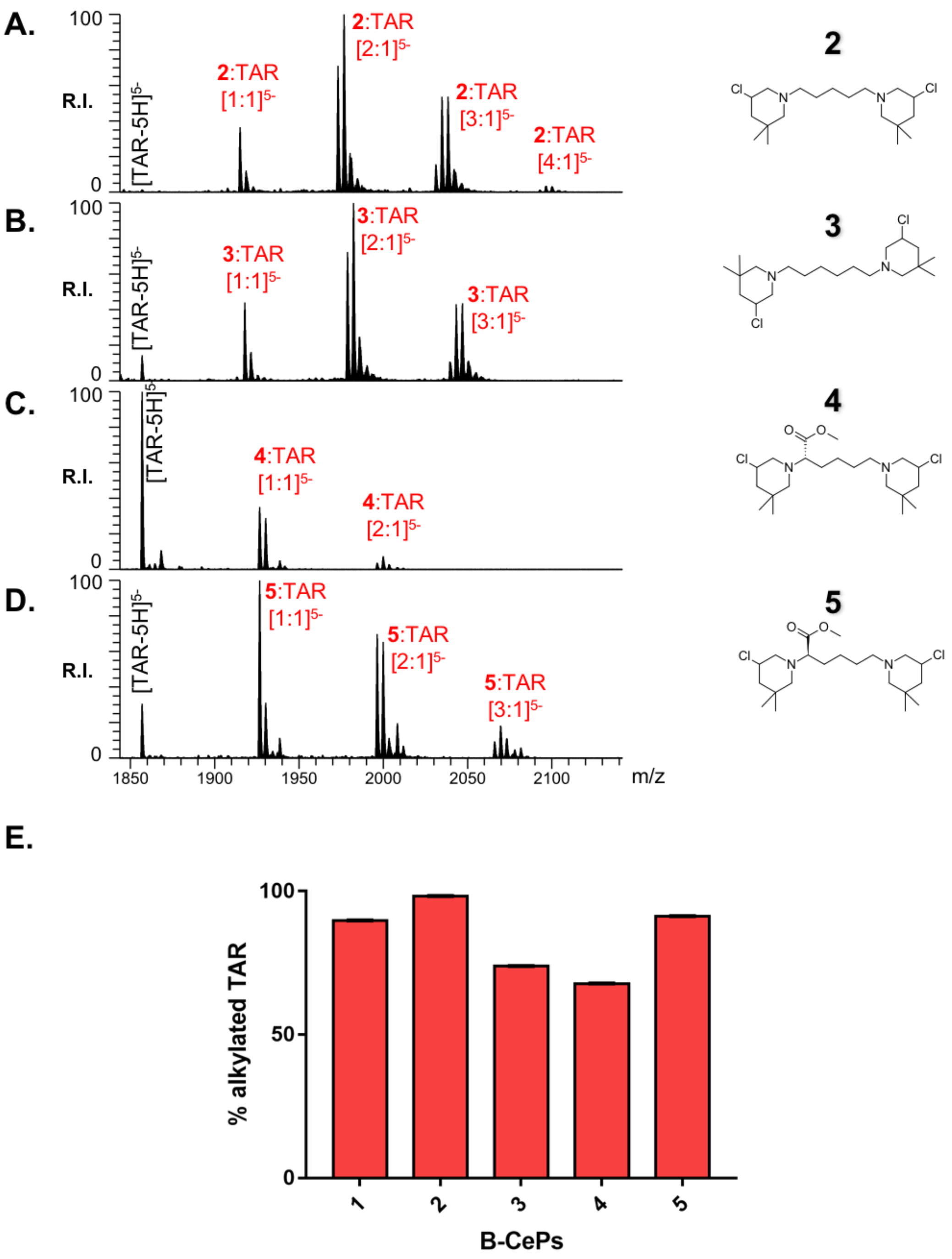

Figure S1. Representative ESI-MS spectra of reaction mixtures obtained by incubating TAR (1 µM) with 10 µM of different B-CePs at 37 °C for 2 h in BPE buffer (pH 7.4). (**A**) was obtained from a sample containing **2** and TAR, (**B**) **3** and TAR, (**C**) **4** and TAR, and (**D**) **5** and TAR. Spectra were recorded in 150 mM ammonium acetate. Lower intensity signals near free/adducted species consist of typical sodium and ammonium adducts. The spectra show the concentration-dependent reactions induced by B-CeP 1 on TAR. Only region containing the 5- charge state is shown. Increasing binding stoichiometries, including combinations of mono- and bi-functional adducts, were detected and indicated in red in the figure. (**E**) Histograms displaying the percentages of alkylated TAR RNA substrate observed in A, B, C, D and Figure 4C for B-CePs **1-5**. The indicated percentages of alkylated substrate provide a measure of the relative reactivity of B-CePs derivatives for the RNA substrate.

Table S1. Digestion products obtained by treating TAR RNA control with RNAse A. Experimental and calculated monoisotopic masses are reported in mass units.

RNA fragments	Sequence	Experimental mass (u)	Calculated mass (u)
G1:C3	GGCp	1013.14	1013.14
G1:U7	GGCAGAUp	2322.30	2322.30
A4:U7	AGAUp	1327.18	1327.18
G10:C13	GAGCp	2684.38	2684.38
G10:C14	GAGCCp	1647.22	1647.22
G10:U15	GAGCCUp	1952.32	1952.32
G16:C21	GGGAGCp	2032.28	2032.28
G16:U22	GGGAGCUp	2338.31	2338.31

Table S2. Digestion products obtained by treating B-CeP **1**-reacted TAR RNA with RNAse A corresponding to unbridged RNA fragments. Experimental and calculated monoisotopic masses are reported in mass units.

Alkylated RNA fragments	Sequence	Experimental mass (u)	Calculated mass (u)
G1:C3 + **1M**	GGCp + **1M**	1293.38	1293.38
G1:U7 + **1M**	GGCAGAUp + **1M**	2602.54	2602.54
A4:U7 + **1M**	AGAUp + **1M**	1607.42	1607.42
G10:C14 + **1M**	GAGCCp + **1M**	1927.46	1927.46
G10:C14 + **1B**	GAGCCp + **1B**	1909.46	1909.46
G10:U15 + **1M**	GAGCCUp + **1M**	2232.56	2232.56
G16:C21 + **1M**	GGGAGCp + **1M**	2312.52	2312.52
G16:C21 + **1B**	GGGAGCp + **1B**	2294.52	2294.52
G16:U22 + **1M**	GGGAGCUp + **1M**	2618.55	2618.55
G16:U22 + **1B**	GGGAGCUp + **1B**	2600.55	2600.55
